# Tumor-suppressive microRNA-218 inhibits tumor angiogenesis via targeting the mTOR component RICTOR in prostate cancer

**DOI:** 10.18632/oncotarget.14131

**Published:** 2016-12-24

**Authors:** Bing Guan, Kaijie Wu, Jin Zeng, Shan Xu, Lijun Mu, Yang Gao, Ke Wang, Zhenkun Ma, Juanhua Tian, Qi Shi, Peng Guo, Xinyang Wang, Dalin He, Yuefeng Du

**Affiliations:** ^1^ Department of Urology, First Affiliated Hospital of Xi’an Jiaotong University, Xi’an, Shaanxi, China; ^2^ Oncology Research Laboratory, Key Laboratory of Environment and Genes Related to Diseases, Ministry of Education, Xi’an, Shaanxi, China

**Keywords:** microRNA-218, prostate cancer, angiogenesis, VEGFA, RICTOR

## Abstract

MicroRNAs, a kind of small non-coding RNAs, can regulate gene expression by targeting mRNAs for translational repression or degradation. Much evidence has suggested that miR-218 was a tumor suppressor in many human cancers including prostate cancer. However, the underlying role of miR-218 in tumor angiogenesis and the mechanisms in PCa and other cancers remains to be unclear. Here in this present study, we demonstrated that miR-218 inhibited the tumor angiogenesis of PCa cells *in vitro* and *in vivo*. RICTOR, the mTOR component 2, was a direct target of miR-218 and miR218-RICTOR-VEGFA axis was the mechanism inhibiting the tumor angiogenesis of PCa cells. RICTOR knockdown phenocopied miR-218 overexpression in inhibiting prostate cancer angiogenesis. Altogether, our findings indicate that down-regulation of miR-218 contributes to tumor angiogenesis through RICTOR/VEGFA axis in PCa, providing new insights into the potential mechanisms of PCa oncogenesis and revealing the potential of miR-218 as a useful serum biomarker and a new therapeutic target for human PCa.

## INTRODUCTION

Prostate cancer (PCa) is the second most frequently diagnosed cancer among men worldwide, with estimated 903,500 new cases and 258,400 deaths per year [1]. In the United States, 220,800 new prostate cancer cases and 27,540 cancer deaths are estimated to occur in 2015 [2]. Most patients with metastatic PCa are initially responsive to androgen-deprivation therapy (ADT) in first treatment. However, a large proportion of cancers eventually become resistant to ADT, and develop to castration-resistant prostate cancer (CRPC), which is the leading cause of death [3]. Therefore, the molecular mechanisms of PCa development and metastasis need further exploration helping to develop effective therapies for this disease.

Micro-RNAs (miRNAs), a new class of small non-coding RNAs, are found to be able to regulate tumor development via modulating both mRNA stability and translation ability into protein [4, 5]. Until now, a significant amount of evidence has suggested that miRNAs are aberrantly expressed in many human cancer types and play important roles in the cancer initiation, development and metastasis [6, 7]. Recent research has reported that several miRNAs may regulate the vascular development, which be vital for tumorigenesis and development [8, 9]. MiR-378 enhances cell survival, tumor growth and angiogenesis through suppressing the expression of two tumor suppressors, Sufu and Fus-1 [10]. MiR-874 suppresses gastric cancer progression by modulating angiogenesis through STAT3/VEGF-A pathway [11]. Conversely, miR-15b, miR-16, miR-20a and miR-20b are potential anti-angiogenic miRNAs through targeting VEGF [12]. Published data shows that miR-218 is a tumor suppressive miRNA in human cancers including prostate cancer. MiR-218 can inhibit cell growth, migration, invasion and cancer stem-like cell self-renewal, and induce apoptosis in cancers [13–15]. However, the role of miR-218 in PCa angiogenesis remains unknown.

In the present study, we found that miR-218 repressed tumor angiogenesis of PCa cells *in vitro* and *in vivo*. RICTOR, the mTOR component 2, was a direct target of miR-218 and miR218/RICTOR axis was the potential mechanism. RICTOR knockdown phenocopied miR-218 overexpression in inhibiting prostate cancer angiogenesis. Therefore, our findings provide new insights into the potential mechanisms of PCa oncogenesis, revealing the potential of miR-218 as a useful biomarker or therapeutic target for PCa.

## RESULTS

### miR-218 is down-regulated in PCa cell lines and tissues

**Figure 1:**
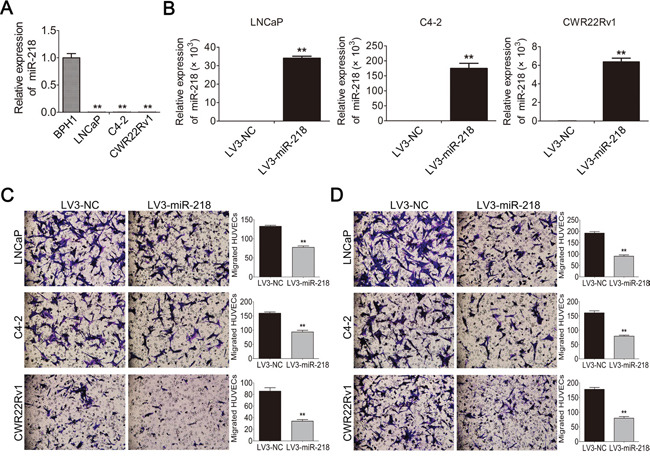
miR-218 is down-regulated in PCa cells and inhibits HUVEC migration in vitro **A**. The expression level of miR-218 was analyzed by miRNA Real-time PCR in a normal prostate epithelial cell line BPH-1 and different PCa cell lines (i.e., LNCaP, C4-2 and CWR22Rv1). U6 was used for normalization. **B**. Stable miR-218 overexpression PCa cell lines were constructed. **C**. Overexpression of miR-218 decreased the recruitment of HUVECs by conditioned medium (CM), which was collected from the LNCaP, C4-2 and CWR22Rv1/LV3-NC or LV3-miR-218 cells. HUVECs were seeded on the upper side of the Transwell insets within 18 hours. Migrated cells in 6 random fields (200 ×) per well were counted. **D**. Overexpression of miR-218 suppressed the recruitment of HUVECs in a co-cultured system. Cancer cells cultured in the bottom of 24-well plate were used to recruit HUVECs seeding on the upper side of the transwell insets. These data were representative of three independent experiments. Asterisks indicate a significant difference compared with controls at *p < 0.05, **p < 0.01.

To determine whether miR-218 is mis-regulated in PCa cell lines, we performed miRNA RT–PCR experiments, and the result showed that several PCa cell lines, including LNCaP, C4-2 and CWR22Rv1, had undetectable or very low level of miR-218 expression compared to normal prostate epithelial cell line BPH-1 (Figure 1A). Moreover, we downloaded the microRNA expression data from GEO database to compare the miR-218 expression level between human PCa samples and their normal adjacent benign prostate tissues. The result showed that the expression level of miR-218 in human PCa tissues was much lower than that in the adjacent normal tissues (Supplementary Figure 1A), especially in metastatic PCA tissues (Supplementary Figure 1B). Overall, these results indicate that miR-218 is down-regulated in both PCa cell lines and tissues.

### miR-218 inhibits tumor angiogenesis of PCa cells *in vitro*

**Figure 2:**
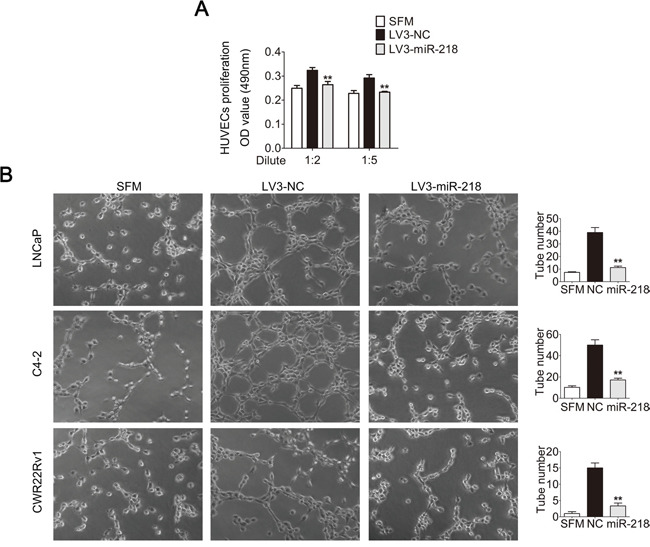
miR-218 inhibits HUVEC proliferation and tube formation in vitro **A**. miR-218 overexpression reduced the proliferation of HUVECs. HUVECs were treated with serum free medium (SFM) or diluted CMs for 48 hours before MTT assay. **B**. miR-218 overexpression suppressed tube formation of HUVECs. HUVECs diluted in SFM or CMs were added into Matrigel-coated wells and incubated for 4 hours. Representative photographs of tube-like structures were taken and tube number in the whole field was counted (right). These data were representative of three independent experiments. *p < 0.05, **p < 0.01 compared with NC group.

To reveal the impact of miR-218 on tumor angiogenesis in PCa, three different PCa cell lines with ectopic miR-218 overexpression were constructed (Figure 1B). The conditioned medium (CM) of these cells was collected, and an *in vitro* endothelial recruitment assay was used to investigate the effects of miR-218 on the migration of human umbilical vein endothelial cells (HUVECs). HUVECs were seeded into upper chambers and different groups of CM were added into the lower chambers as attraction. After incubation for 18 hours, the migration of HUVECs was suppressed by miR-218 overexpression in LNCaP, C4-2 and CWR22Rv1 cells (Figure 1C). In a co-cultured system, the migration of HUVECs was abolished by miR-218 overexpression in LNCaP, C4-2 and CWR22Rv1 cells as well (Figure 1D). Furthermore, the MTT assay was also performed to assess the effects of different groups of CM on the proliferation of HUVECs. In consistency, it was found that CM from LNCaP and C4-2 cells transfected with miR-218 caused a decrease of HUVECs proliferation compared with the negative control groups (Figure 2A). Moreover, the tube-formation assay with HUVECs was performed. The formation of tube-like structures in Matrigel was also suppressed by miR-218 overexpression in LNCaP, C4-2 and CWR22Rv1 cells compared with the control groups (Figure 2B). Therefore, these results suggest that overexpression of miR-218 inhibits the process of tumor angiogenesis of PCa cells.

### VEGFA expression is inhibited by miR-218 in PCa cells

**Figure 3:**
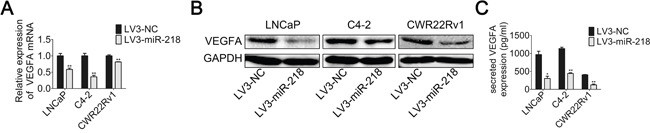
miR-218 inhibits VEGFA expression **A**. Real-time PCR was used to analyze the expression level of VEGFA in LNCaP, C4-2 and CWR22Rv1 cells transfected with LV3-NC or LV3-miR-218. **B**. VEGFA protein level were analyzed by Western blotting analysis. **C**. Concentration of secreted VEGFA protein in the CMs was determined by ELISA. These data were representative of three independent experiments. *p < 0.05, **p < 0.01 compared with NC group.

Next, the expression level of VEGFA, a most important angiogenic factor in vasculature and angiogenesis, was tested. Compared with the control groups, VEGFA mRNA level in all PCa cell lines with miR-218 overexpression decreased (Figure 3A). Similarly, the protein level of VEGFA also decreased (Figure 3B). ELISA assay was also used to detect secreted VEGFA protein level in the supernatant of above cell lines. As expected, overexpression of miR-218 also inhibited the secreted VEGFA protein level (Figure 3C).

### miR-218 targets RICTOR by binding to its 3’-UTR

**Figure 4:**
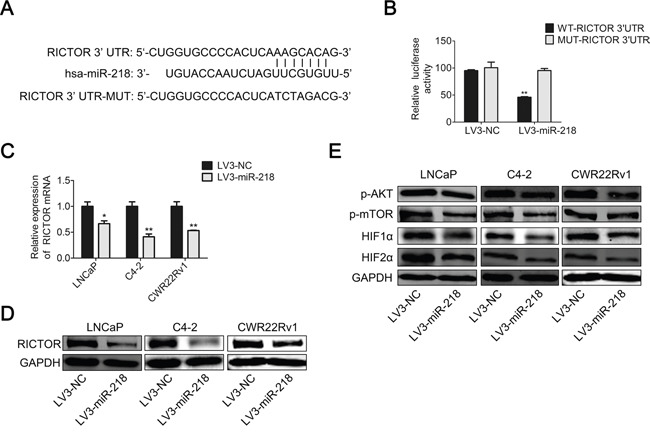
miR-218 inhibits VEGFA expression by targeting RICTOR 3’-UTR **A**. RICTOR 3’-UTR regions containing the wild-type or mutant binding site and the sequence complementarity between miR-218 and the RICTOR 3’-UTR (WT and MUT) were shown. **B**. Relative luciferase activity was analyzed after transfection with the wild-type or mutant 3’-UTR reporter plasmids in LV3-NC or LV3-miR-218 293T cells. Overexpression of miR-218 suppressed luciferase activity of the wild-type reporter (50%). **C**. Real-time PCR was used to analyze the expression level of RICTOR in LNCaP, C4-2 and CWR22Rv1 cells transfected with LV3-NC or LV3-miR-218. **D**. RICTOR protein level was analyzed by Western blotting analysis. E. The protein level of phosphorylation status of AKT at Ser-473, p-mTOR, HIF1α and HIF2α were analyzed by Western blotting analysis. GAPDH was used as an internal control. The results were representative of three independent experiments. The values were shown as the mean ± SD. *p < 0.05, **p < 0.01.

TargetScan (http://www.targetscan.org/) was used to predict genes which were targeted by miR-218, and we identified a putative miR-218 binding site within the 3’-UTR of RICTOR (Figure 4A). In order to confirm the direct targeting and binding between miR-218 and the 3’-UTR of RICTOR, a dual-luciferase reporter assay was performed. The wild-type (WT) and mutant (MUT) versions of the RICTOR 3’-UTR containing site-directed mutations in the putative miR-218 target sites, were cloned into the reporter plasmids. Overexpression of miR-218 significantly suppressed the luciferase activity of wild-type reporter (50%) but not mutant reporter (Figure 4B). This suggests that the 3’-UTR of RICTOR is specifically targeted by miR-218.

### miR-218 suppresses RICTOR/mTOR/HIF1α&HIF2α/VEGFA axis

In addition, overexpression of miR-218 in PCa cell lines, including LNCaP, C4-2 and CWR22Rv1, resulted in the down-regulation of RICTOR both at mRNA level and protein level compared with the negative control (Figure 4C and 4D). Also, the phosphorylation status of AKT at Ser-473, p-mTOR, HIF1α and HIF2α decreased in the miR-218-overexpressed PCa cells at the protein level (Figure 4E). These results indicate that miR-218 may suppress RICTOR/mTOR/HIF1α&HIF2α/VEGFA axis, and then inhibits tumor angiogenesis of PCa cells.

### miR-218 inhibits tumor growth and angiogenesis of PCa cells *in vivo*

**Figure 5:**
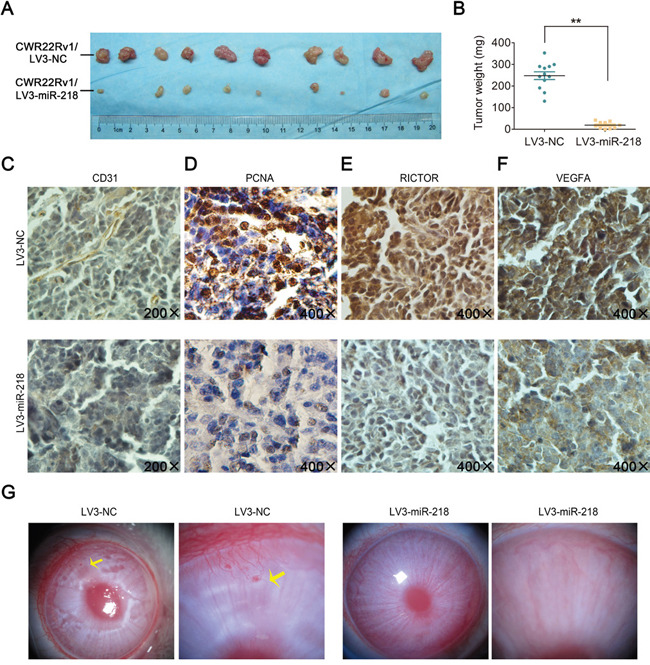
miR-218 inhibits tumor growth and angiogenesis *in vivo* **A**. Subcutaneous xenografts of CWR22Rv1/LV3-NC and CWR22Rv1/LV3-miR-218 subclones were harvested 2 weeks after inoculation. **B**. Tumor weights between the two groups. The values were shown as the mean ± SD. *p < 0.05, **p < 0.01. **C**-**F**. The expression of CD31, PCNA, RICTOR and VEGFA were analyzed in paraffin fixed tumor sections from CWR22Rv1/LV3-NC and CWR22Rv1/LV3-miR-218 xenografts by immunohistochemistry; representative photographs were shown at magnification × 200 (CD31) or × 400 (PCNA, RICTOR, VEGFA). **G**. CWR22Rv1/LV3-NC cells but not CWR22Rv1/LV3-miR-218 cells could induce angiogenesis in rabbit cornea (n = 3). Neonatal vessels invaded the cornea, connecting the tumor (yellow arrow) and the cornea limbal vascular plexus.

To confirm the effects of miR-218 on tumorigenicity *in vivo*, transfected CWR22Rv1 cells were injected into the flanks of nude mice to form xenograft tumors. A slower tumor growth in the CWR22Rv1/LV3-miR-218 group was observed (Figure 5A). The average tumor weight from the CWR22Rv1/LV3-miR-218 group was lower than that from the control group (Figure 5B). Moreover, CD31 (endothelial cell marker) in those CWR22Rv1 xenograft tissues was stained by immunohistochemistry (IHC) assay, and fewer CD31-positive tumor tissues in CWR22Rv1/LV3-miR-218 group was observed than that from the control group (Figure 5C). Consistently, similar results were observed in the staining of proliferating cell nuclear antigen (PCNA), RICTOR and VEGFA in these tumor tissues (Figure 5D-5F). Furthermore, a more specific and sensitive rabbit cornea assay was performed to verify its role of angiogenesis inhibition. Three weeks after miR-218-transfected CWR22Rv1 cells transplanted into rabbit cornea, CWR22Rv1/NC cells induced a neovascular response and formed visible tumors, but CWR22Rv1/LV3-miR-218 cells lost these abilities (Figure 5G). Taken together, it is found that miR-218 can inhibit tumor growth and angiogenesis *in vivo*.

### RICTOR is critical for angiogenesis in PCa cells

**Figure 6:**
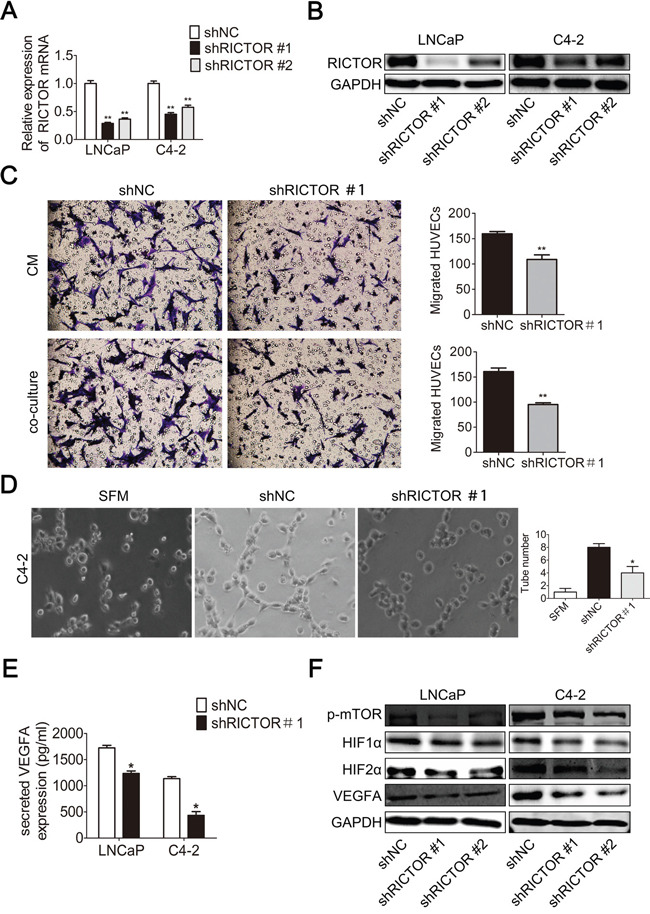
RICTOR knockdown reduces angiogenesis in prostate cancer cells **A**-**B**. Real-time PCR and Western blotting analysis were used to confirm the knockdown of RICTOR in LNCaP and C4-2 cells transfected with LV3-shRICTOR#1/#2 both at the mRNA and protein level. **C**. RICTOR knockdown decreased the recruitment of HUVECs. Conditioned mediums (CMs) collected from the C4-2/LV3-shNC and C4-2/LV3-shRICTOR#1 cells or these cancer cells cultured in the bottom of 24-well plate were used to recruit HUVECs. Migrated cells in 6 random fields (200 ×) per well were counted. **D**. RICTOR knockdownreduced tube formation of HUVECs diluted in SFM or CMs. Representative photographs of tube-like structures were taken and tube numbers in the whole field were counted. **E**. Concentration of secreted VEGFA protein in the CMs was determined by ELISA. **F**. The protein level of p-mTOR, HIF1α, HIF2α and VEGFA were analyzed by Western blotting analysis. GAPDH was used as an internal control. These data were representative of three independent experiments. The values were shown as the mean ± SD. *p < 0.05, **p < 0.01.

To further demonstrate that miR-218 in PCa cells affects the angiogenesis of HUVECs through the regulation of RICTOR, RICTOR knockdown PCa cell lines were constructed. The efficacy of RICTOR knockdown was confirmed both at the mRNA and protein level (Figure 6A-6B). Indeed, the down-regulation of RICTOR was accompanied by a significant reduction of HUVEC migration and tube-formation capacities (Figure 6C-6D). Also, the ELISA assay and WB assay showed that knockdown of RICTOR inhibited mTOR/HIF1α&HIF2α/VEGFA axis (Figure 6E-6F), which was similar with the effects of miR-218 overexpression in PCa cells. These data indicate that miR-218 may inhibit tumor angiogenesis via suppressing RICTOR/mTOR/HIF1α&HIF2α/VEGFA axis in PCa cells.

## DISCUSSION

Despite increasing advances in the study of prostate cancer, there are a number of challenges yet to be overcome in the management of this disease. For example, it remains a problem to determine which patients truly require treatment and which can be safely observed. So it may eventually be possible to identify several miRNAs or molecules that can be measured in serum or tissues for better diagnosis or precise treatment in future [16, 17].

MicroRNAs (miRNAs), a kind of short (18-22 nucleotide), stable and small non-coding RNAs, were first found in nematodes in 1993 [18]. To date, over 1000 miRNA have been identified in human [19]. It is becoming clear that miRNAs play an important role in the regulation of gene expression by targeting mRNAs for translational repression or degradation, and are involved in many cancer biological processes, such as tumor initiation, development, metastasis and angiogenesis. Many miRNAs have been shown to act as tumor suppressors, and the decreased activity of tumor-suppressive miRNAs leads to increased oncogene translation and tumor formation [20]. For example, microRNA-34a inhibits prostate cancer stem cells and metastasis by directly repressing CD44 [21], while miR-203 suppresses tumor growth and angiogenesis by targeting VEGFA in cervical cancer [22].

Many researches have reported that miR-218 acts as a tumor suppressor in kinds of human cancers. MiR-218 is downregulated and inhibits the invasion and metastasis of gastric cancer by targeting the Robo1 receptor [23]. MiR-218 suppresses nasopharyngeal cancer progression and restoring miR-218 expression in NPC might be useful for the clinical management [24]. MiR-218/survivin axis inhibits cervical cancer progression by regulating clonogenicity, migration, and invasion [25]. In addition, miR-218 also suppresses prostate cancer development as well. MiR-218 was significantly overexpressed in all localized high GS and pT3 PCa as well as metastatic carcinoma, suggesting that miR-218 may be involved in the process of metastases of PCa [26]. Also, MiR-218 was significantly downregulated in PCa clinical specimens and loss of tumor-suppressive miR-218 enhanced cancer cell migration and invasion in PCa through direct regulation of LASP1 [27]. Moreover, through repressing TPD52 expression, miR-218 could inhibit prostate cancer growth and promote apoptosis [28].

In addition to the behaviors as described above, tumor angiogenesis is also important for tumor progression. However, the underlying role of miR-218 in tumor angiogenesis of PCa remains unclear. Therefore, we suppose that miR-218 may also inhibit tumor angiogenesis in PCa. As we known, tumor angiogenesis is crucially dependent on the communication between the tumor and the associated endothelium. The migration, proliferation and tube formation of endothelial cells (ECs) are important processes for tumor angiogenesis [29]. In the present study, we find that miR-218 overexpression in PCa cells inhibits HUVEC proliferation, migration and tube formation *in vitro* and suppresses tumor angiogenesis in the xenografts and rabbit corneas.

Mechanistically, miR-218 exerts these functions by down-regulating expression of VEGFA, which is the most prominent factor among the angiogenic cytokines [30–32]. Also, further experiments reveal the mechanisms how miR-218 affects VEGFA. Firstly, we search the potential targets of miR-218 using TargetScan. Among the candidate target genes, we focus on RICTOR, the mTOR component 2. Indeed, we demonstrate that miR-218 negatively regulates RICTOR by binding to a specific target site within the 3’-UTR of RICTOR. Also, we find that overexpression of miR-218 in human PCa cell lines inhibits RICTOR at both mRNA and protein level. In addition, the phosphorylation status of AKT at Ser-473, mTOR, HIF1α, HIF2α and VEGFA expression are decreased in miR-218-overexpressing PCa cells at the protein level. Our findings are consistent with several published reports [13, 33, 34].

**Figure 7:**
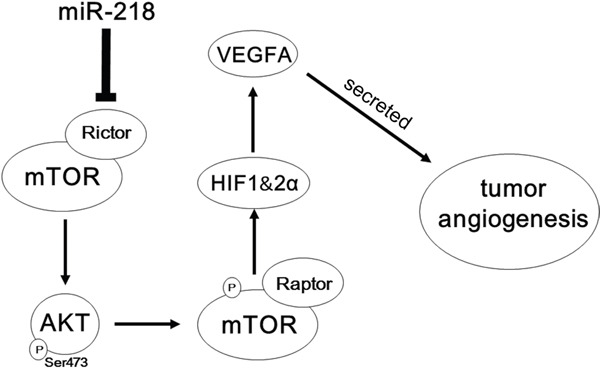
Schematic representation of the roles of miR-218-RICTOR/pAKT/pmTOR-HIF1α&HIF2α-VEGFA signaling on the angiogenic properties of PCa cells

Therefore, our data here show that miR-218 can inhibit angiogenesis through targeting RICTOR/VEGFA axis in PCa (Figure 7). However, the regulation of angiogenesis-related cytokines in human cancer cells is very complex, so we could not rule out the possibility that other signaling pathways modulating VEGF expression may also be affected by miR-218.

In conclusion, we demonstrate that miR-218 suppresses PCa progression by modulating angiogenesis through the RICTOR/VEGFA axis. Also, these findings indicate that miR-218 can be used as a biomarker for diagnosis and treatment of prostate cancer in the future, and miR-218-based molecular targeting therapy may be another useful therapeutic strategy for prostate cancer.

## MATERIALS AND METHODS

### Cell lines and cell culture

Human PCa cell lines LNCaP, C4-2 and CWR22Rv1 were obtained from the American Type Culture Collection (ATCC; Rockville, MD, USA). The umbilical vein endothelial cell line HUVEC and 293T cell line, were kindly provided by Dr. Jer- Tsong Hsieh (University of Texas Southwestern Medical Center, Dallas, TX, USA). The HUVEC and 293T cells were cultured in DMEM medium with 10% fetal bovine serum and LNCaP, C4-2 and CWR22Rv1 cells were cultured in RPMI-1640 medium with 10% fetal bovine serum. All cell lines were cultured in a humidified chamber at 37°C with 5% CO2.

### Bioinformatics

The microRNA expression data for human primary and metastatic PCa samples along with their adjacent benign prostate tissues from a GEO DataSets (GSE21036) was downloaded from GEO website (http://www.ncbi.nlm.nih.gov/geo/) at August 7, 2015 for further analysis.

### Total RNA isolation and reverse transcription

Cells were harvested and total RNA was extracted using Trizol reagent (Life Technologies, Rockville, MD, USA) according to the manufacturer's protocol. The total RNA was reverse-transcribed to cDNA using PrimeScript™ RT reagent kit (Takara, Dalian, China) following the manufacturer's protocol.

### miRNA isolation and reverse transcription

Total RNA was extracted as above. Then the total miRNA was reverse transcribed using the miScript II RT Kit (QIAGEN, Germany) according to the manufacturer's instructions.

### Real-time quantitive PCR (qPCR) assay

The cDNA was studied by CFX96 real-time PCR system (Bio-Rad, Hercules, CA) using SYBR Green PCR Master Mix (Takara, Dalian, China) to detect the transcriptional expression of specific genes (primer sequences shown in Supplementary Table 1). GAPDH or U6 was used for normalization. Relative gene expression was calculated using the 2^-ΔΔCt^ method.

### Lentivirus preparation and infection

The commercial LV3-miR-218 lentivirus vector (pre-miR-218) (containing green fluorescent protein GFP and the puromycin sequence) were constructed by GenePharma (Shanghai, China). An LV3 scrambled lentiviral vector (miR-NC) was used as a negative control. The lentiviral vectors were verified by DNA sequencing. Then the lentiviral vectors were used to infect LNCaP, C4-2 and CWR22RV1 cells at an appropriate multiplicity of infection (MOI) when cells had grown to 40–50% confluence in the presence of 8 μg/ml polybrene. The stable miR-218 overexpression subclones were maintained by using 2–3 μg/ml puromycin-resistant culturing (puromycin, Sigma, USA). After that, cells were analyzed for miR-218 expression using the miRNA real-time quantitive PCR (qPCR) assay described as above.

Lentiviral vectors encoding short hairpin RNA (shRNA) targeting human RICTOR were constructed by GenPharma (Shanghai, China). The specific sequences of shRNA used for targeting human RICTOR were: 5′-GCCGTATACTCCTTCGCAAAG-3′ (#1) and 5′- GCAACCAACTGAGTGCAATAT -3′ (#2). The scrambled lentiviral vector was used as a negative control. The cells were transfected with the lentiviral vectors as above. Afterward, cells were analyzed using real-time quantitive PCR (qPCR) and Western blotting assay for RICTOR expression.

### Conditioned medium collection

Prostate cancer cells were seeded at 8 × 10^5^ per 60 mm culture dish. After adhesion, cells were washed with serum free medium (SFM) for three times, fed with 3 ml SFM and cultured for 24 hours. Then the supernatant was collected and centrifuged to remove cell debris. Conditioned mediums (CMs) were stored in -80°C before use.

### HUVEC migration assay

Transwell migration assays were performed in 8-μm-pore transwell inserts (Millipore, Bedford, MA, USA). 400 μl HUVECs suspended in SFM at 1.5 × 10^5^ cells/ml was seeded into upper chamber and 1 ml of different conditioned mediums was added to the lower chamber as a chemoattractant. After incubation for 18 hours, the upper surface of the insert was wiped with cotton swab and cells migrated to the lower surface were fixed by 4% paraformaldehyde and stained with crystal violet. Cell number was counted in 6 random fields per well (200 ×).

### Tube formation assay

HUVECs (1 × 10^5^/well) suspended in SFM or SFM diluted (1:1) conditioned medium were seeded into Matrigel-coated wells in a 24-well plate. Four hours later, photographs were taken. Only perfectly continuous tubes between 2 branching points were considered as a tube.

### MTT assay

Growth rates of cells were measured by 3-(4, 5-dimethylthiazol-2-yl)-2, 5-diphenyltetrazolium bromide (MTT) assay. HUVECs (3×10^3^/well) were seeded in 96-well culture plates and incubated overnight. Then, the cells were treated with conditioned mediums for 2 days. After being washed once, 0.5 mg/ml of MTT was added and incubation was carried out at 37°C. Four hours later, the culture medium was removed carefully and dimethyl sulfoxide (DMSO) was added to solubilize the formazan crystals. Finally, the absorbance was measured at a wavelength of 490 nm using a microplate autoreader (BioTek Instruments Inc., Winooski, VT, USA). Independent experiments were repeated in triplicate.

### ELISA assay

The protein level of VEGFA in CMs was measured using RayBio^®^ Human VEGF ELISA Kit (RayBiotech Inc, Norcross, GA, USA) according to the manufacturer's instructions.

### Western blotting assay

Cells were washed three times using cold PBS and lysed in RIPA buffer (50 mM Tris pH 8.0, 150 mM NaCl, 0.1% SDS, 1% NP-40, and 0.5% sodium deoxycholate) containing protease inhibitors. Approximate 30 μg of protein was separated with 10% SDS–PAGE gel and blotted onto nitrocellulose membranes. Then membranes were blocked with 5% skim milk at room temperature for 1 hour and then incubated with primary antibodies against GAPDH (Shanghai Kangchen), RICTOR (Bethyl Laboratories), VEGFA (Abcam), Akt (Cell signaling Technology), mTOR (Cell signaling Technology), HIF1α (Abcam) and HIF2α (Abcam) at 4°C overnight, followed by TBST wash and 1 hour incubation with horseradish peroxidase-conjugated secondary antibodies at room temperature. Protein bands were visualized by a Molecular Imager ChemiDoc XRS System (Bio-Rad Laboratories, Hercules, CA, USA).

### Dual luciferase activity assay

The 3’UTR of the RICTOR mRNA containing either the putative or mutated miR-218 binding site was synthesized by GENECHEM (Shanghai, China). This sequence was cloned into the XbaI/XbaI restriction sites of the GV272 luciferase reporter vector (GENECHEM, China) to generate the RICTOR 3’UTR reporter. A total of 8 × 10^4^ 293T cells stably transfected with pre-miR- 218 or miR-NC was seeded into 24-well plates. To perform dual luciferase activity assay, ERE-TK-Luc and GV272-WT-RICTOR/GV272-MUT-RICTOR 3’UTR reporter plasmid were co-transfected into 293T sublines using X-tremeGENE HP DNA transfection reagent (Roche, Mannheim, Germany). Luciferase assay was carried out using the Dual Luciferase Assay kit (Promega, Madison, WI, USA) following the manufacturer's instructions. Three wells of cells were used for each data point.

### Xenograft tumor model and rabbit cornea assay

For tumorigenesis assay in nude mice, 8 × 10^6^ cells were injected subcutaneously into both sides of the flank region. Five mice were used for each subclone. Xenograft tumors were harvested, weighted and fixed with 4% paraformaldehyde after 2 weeks. For rabbit assay in New Zealand white rabbits, a micropocket (1.5 × 3 mm) was surgically produced in the 6 and 12 o’clock points of the right eye. 1.5 × 10^5^ CWR22Rv1/pre-miR-218 or miR-NC cells were implanted into the micropockets (four rabbits per group). After 3 weeks, angiogenic responses in corneas were evaluated. Animal care and protocols were in accordance with the guidelines of the Institutional Animal Care and Use Committee of Xi’an Jiaotong University [35].

### Immunohistochemistry

The nude mice xenografts specimens were studied by immunohistochemistry (IHC) assay using EnVisionTM System (DAKO, Carpinteria, CA, USA), according to the manufacturer's instructions. Primary antibodies used in IHC were CD31 (Abcam, ab28364, 1:50), PCNA (Proteintech, 10205-2-AP, 1:200), RICTOR (Bethyl Laboratories, A300-459A, 1:200), VEGF-A (Abcam, ab46154, 1:150). Staining signals were photographed using an Olympus BX51 Microscope (Olympus, Tokyo, Japan). Average intensity score of the positive cells (0 -none, 1-weak, 2-intermediate, and 3-strong) and percentage score of stained cells (1–0% to 25%, 2–25% to 50%, 3–50% to 75%, and 4–75% to 100%) were multiplied to get the total staining score, ranging from 0 to 12. Three fields were selected for examination.

### Statistical analysis

GraphPad Prism version 6.0 software (GraphPad, San Diego, CA, USA) was used to analyze differences between two groups (student's *t*-test), calculate Pearson's correlation and perform linear regression analysis. A *p* value less than 0.05 was considered to be significant.

## SUPPLEMENTARY MATERIALS FIGURES AND TABLES


