# Correction: Tumor-suppressive microRNA-218 inhibits tumor angiogenesis via targeting the mTOR component RICTOR in prostate cancer

**DOI:** 10.18632/oncotarget.28206

**Published:** 2022-03-07

**Authors:** Bing Guan, Kaijie Wu, Jin Zeng, Shan Xu, Lijun Mu, Yang Gao, Ke Wang, Zhenkun Ma, Juanhua Tian, Qi Shi, Peng Guo, Xinyang Wang, Dalin He, Yuefeng Du

**Affiliations:** ^1^Department of Urology, First Affiliated Hospital of Xi’an Jiaotong University, Xi’an, Shaanxi, China; ^2^Oncology Research Laboratory, Key Laboratory of Environment and Genes Related to Diseases, Ministry of Education, Xi’an, Shaanxi, China; ^*^These authors have contributed equally to this work

**This article has been corrected:** In Figure 2B, the C4-2 image in the ‘LV3-miR-218’ column contains an accidental duplication of the CWR22Rv1 image in the same column. The corrected Figure 2, produced using the original data, is shown below. The authors declare that these corrections do not change the results or conclusions of this paper.

Original article: Oncotarget. 2017; 8:8162–8172. 8162-8172. https://doi.org/10.18632/oncotarget.14131

**Figure 2:**
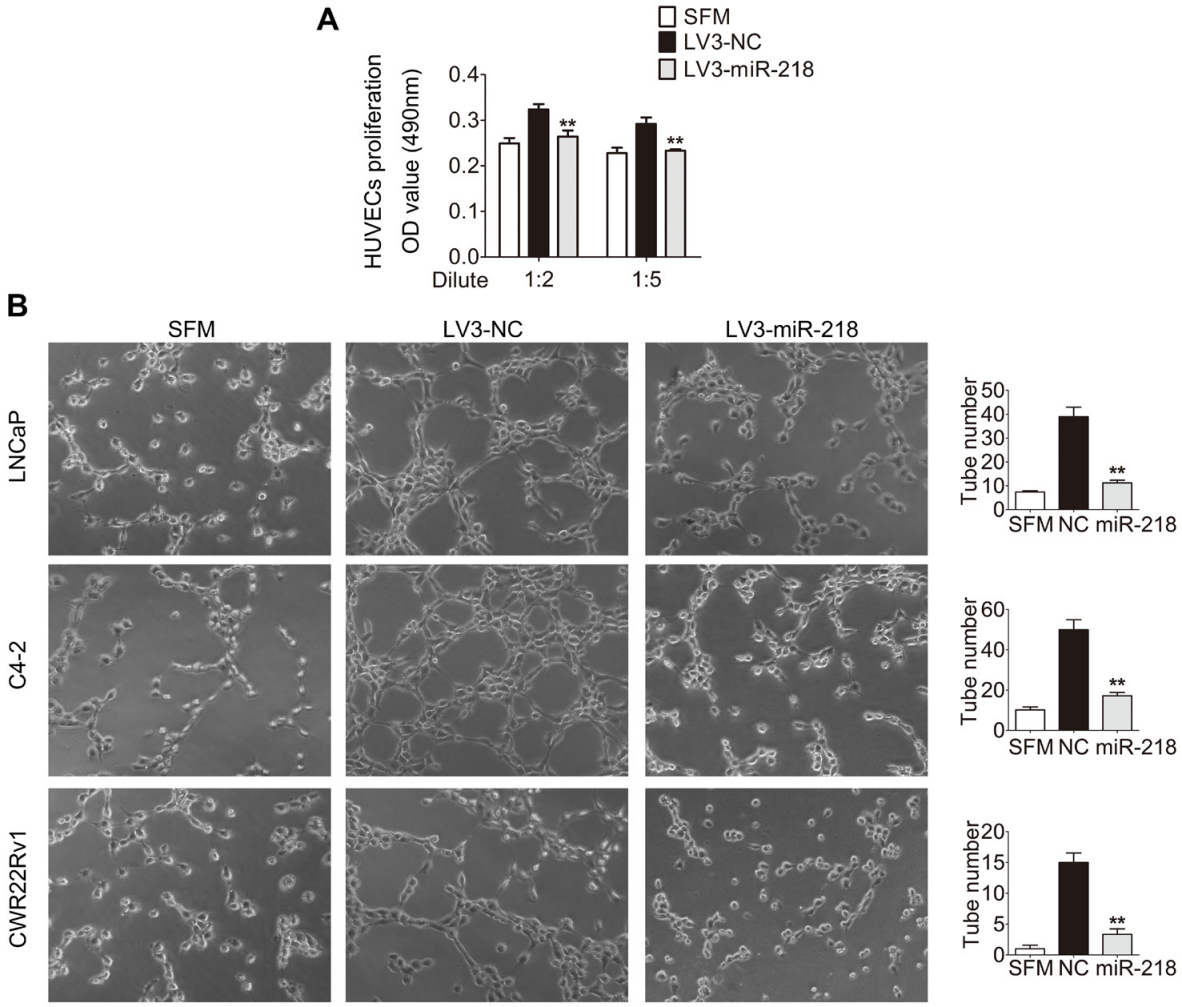
miR-218 inhibits HUVEC proliferation and tube formation *in vitro*. (**A**) miR-218 overexpression reduced the proliferation of HUVECs. HUVECs were treated with serum free medium (SFM) or diluted CMs for 48 hours before MTT assay. (**B**) miR-218 overexpression suppressed tube formation of HUVECs. HUVECs diluted in SFM or CMs were added into Matrigel-coated wells and incubated for 4 hours. Representative photographs of tube-like structures were taken and tube number in the whole field was counted (right). These data were representative of three independent experiments. ^*^ *p* < 0.05, ^**^ *p* < 0.01 compared with NC group.

