# Supplementary material for: Tumor-suppressive microRNA-218 inhibits tumor angiogenesis via targeting the mTOR component RICTOR in prostate cancer
Source: Oncotarget. 2016 Dec 24;8(5):8162–72. doi: 10.18632/oncotarget.14131 (PMC5352391; doi:10.18632/oncotarget.14131)
Supplement: Supplementary file 1 [file oncotarget-08-8162-s001.pdf]

Tumor-suppressive microRNA-218 inhibits tumor angiogenesis via targeting the mTOR component RICTOR in prostate cancer

SUPPLEMENTARY FIGURE AND TABLE

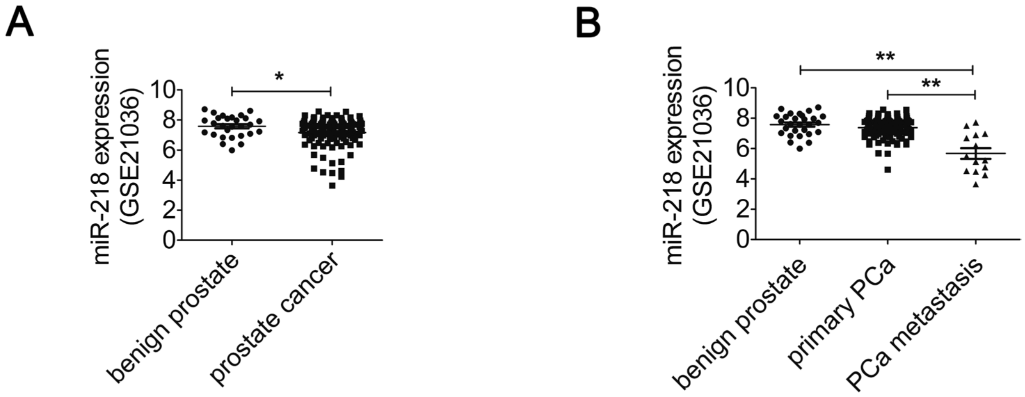

Supplementary Figure 1: miR-218 is down-regulated in human prostate cancer tissues. A. The expression data of miR-218 in human PCa tissues and the adjacent normal tissues downloaded from a GEO datasets was analyzed. **B.** Expression of miR-218 in human primary and metastatic prostate cancer samples and control normal adjacent benign prostate. Asterisks indicated a significant difference compared with controls at *$p < 0.05$, **$p < 0.01$.

Supplementary Table 1: Primer sequences used in real-time qPCR

Gene	Forward primer	Reverse primer
GAPDH	ATGGGGAAGGTGAAGGTCGG	GACGGTGCCATGGAATTTGC
RICTOR	GCTAGGTGCATTGACATACAACA	AGTGCTAGTTCACAGATAATGGC
VEGFA	GAGCCTTGCCTTGCTGCTCTA	CACCAGGGTCTCGATTGGATG
